# High-Throughput Ultrasensitive Molecular Techniques for Quantifying Low-Density Malaria Parasitemias

**DOI:** 10.1128/JCM.01057-14

**Published:** 2014-09

**Authors:** Mallika Imwong, Sarun Hanchana, Benoit Malleret, Laurent Rénia, Nicholas P. J. Day, Arjen Dondorp, Francois Nosten, Georges Snounou, Nicholas J. White

**Affiliations:** a Department of Molecular Tropical Medicine and Genetics, Faculty of Tropical Medicine, Mahidol University, Bangkok, Thailand; b Mahidol Oxford Research Unit, Faculty of Tropical Medicine, Mahidol University, Bangkok, Thailand; c Laboratory of Malaria Immunobiology, Singapore Immunology Network (SIgN), Agency for Science Technology and Research (A*STAR), Biopolis, Singapore; d Department of Microbiology, Yong Loo Lin School of Medicine, National University of Singapore, National University Health System, Singapore; e Centre for Tropical Medicine, Nuffield Department of Medicine, Churchill Hospital, Oxford, United Kingdom; f Shoklo Malaria Research Unit, Mahidol-Oxford Tropical Medicine Research Unit, Faculty of Tropical Medicine, Mahidol University, Mae Sot, Thailand; g Sorbonne Universités, UPMC University Paris 06, UPMC UMRS CR7, Paris, France; h Centre d'Immunologie et de Maladies Infectieuses (CIMI)–Paris, Institut National de la Santé et de la Recherche Médicale (INSERM) U1135–Centre National de la Recherche Scientifique (CNRS) ERL 8255, Paris, France

## Abstract

The epidemiology of malaria in “low-transmission” areas has been underestimated. Molecular detection methods have revealed higher prevalences of malaria than conventional microscopy or rapid diagnostic tests, but these typically evaluate finger-prick capillary blood samples (∼5 μl) and therefore cannot detect parasite densities of <200/ml. Their use underestimates true parasite carriage rates. To characterize the epidemiology of malaria in low-transmission settings and plan elimination strategies, more sensitive quantitative PCR (qPCR) is needed to identify and quantify low-density malaria parasitemias. A highly sensitive “high-volume” quantitative PCR (qPCR) method based on Plasmodium sp. 18S RNA was adapted for blood sample volumes of ≥250 μl and scaled for high throughput. The methods were validated by assessment of the analytical sensitivity and specificity, diagnostic sensitivity, and specificity, efficiency, precision, analytical and diagnostic accuracies, limit of detection, root cause analysis of false positives, and robustness. The high-volume qPCR method based on Plasmodium sp. 18S RNA gave high PCR efficiency of 90 to 105%. Concentrations of parasite DNA from large volumes of blood gave a consistent analytical detection limit (LOD) of 22 parasites/ml (95% CI, 21.79 to 74.9), which is some 2,500 times more sensitive than conventional microscopy and 50 times more sensitive than currently used PCR methods from filter paper blood spots. The diagnostic specificity was 99.75%. Using automated procedures it was possible to process 700 blood samples per week. A very sensitive and specific high-throughput high-volume qPCR method for the detection of low-density parasitemias (>20 parasites/ml) was developed and validated.

## INTRODUCTION

Elimination of malaria, the most important parasitic disease of humans, is now firmly back on the global agenda. Major reductions in malaria morbidity and mortality have been observed recently, but these gains and the goal of elimination are threatened by the emergence of artemisinin-resistant Plasmodium falciparum in South-East Asia. P. falciparum coexists in Asia with Plasmodium vivax, which is also developing resistance to current treatments and is intrinsically more difficult to eliminate because of its dormant liver stages. The spread of artemisinin resistance in P. falciparum, which is now compromising the therapeutic efficacy of artemisinin combination treatments (ACTs), might derail current global initiatives to control and eliminate malaria (1). Consensus is growing that elimination of falciparum malaria in regions with artemisinin resistance is the only effective approach to counter this threat (2, 3). In order to eliminate malaria, it is essential to define its epidemiology. Conventional descriptions of the epidemiology of malaria in low-transmission settings suggest that malaria prevalences are low (<10%) and heterogeneous. Most or all infections have been thought to be symptomatic, since the population is considered to remain nonimmune in these low-transmissions settings, so the focus of malaria control activities has been on the identification and treatment of symptomatic individuals.

Molecular assays to detect Plasmodium infections have been increasingly implemented because, although they are not point of care, they have higher sensitivity than microscopy or rapid diagnostic tests (RDTs), and they also provide definitive species identification and additional information on genotype. PCR for malaria detection has been developed based on several different target genes such as 18S RNA (4–11), tRNA (12), and mitochondrial genes such as cytochrome *b* (13, 14), AMA1 (15), and Stevor (16). Of these, the 18S RNA gene has been most commonly used as the target of PCR malaria detection because it is specific, conserved in all Plasmodium species, and has ∼5 to 7 copies per genome, which increases sensitivity (17). The detection level (sensitivity) of these PCR methods, either as nested PCR or real-time PCR, is usually in the range of 100 to 1000 parasites per milliliter (18). Sensitive PCR methods reveal a high prevalence of low-density parasitemia in areas of endemicity (5, 6), but nearly all epidemiological studies reported to date applied the PCR assay on small-volume blood samples (5, 6, 18–23), typically finger-prick capillary blood samples (≤5 μl). These have also been used in “focused screening and treatment” strategies (24). Even if it were possible to reliably amplify the DNA from a single parasite, the sensitivity of this method is still limited by the sample volume to ≥1 parasite/5 μl, so that parasite densities of <200/ml cannot be detected. In reality, with the use of capillary blood samples, the limit of detection is around 1,000 or more parasites per ml. Larger blood volume PCR detection has been reported but not widely used (19).

To detect and quantify low-density malaria parasitemias in large sample sets, we developed a highly sensitive quantitative PCR (qPCR) based on a previously published methodology (11) adapted for a blood sample volume of ≥200 μl. The method was validated by assessment of the analytical sensitivity and specificity, diagnostic sensitivity and specificity, efficiency, precision, analytical and diagnostic accuracies, limit of detection, and robustness. It was then adapted for high throughput use in order to facilitate epidemiological screening in areas where malaria elimination activities might be implemented.

## MATERIALS AND METHODS

### Blood sample collection and DNA extraction.

Whole blood samples (1 ml) were collected in EDTA tubes and centrifuged at 2,500 rpm for 10 min to remove all plasma together with 70 to 100 μl of the buffy coat per 1 ml of whole blood. The packed red blood cells (RBCs) were then frozen at −30°C. The DNA template for PCR detection and quantification of Plasmodium was purified from the thawed packed red blood cell samples. After careful measurement of sample volume, DNA was extracted using a QIAamp blood minikit for sample volumes of ≤200 μl (equivalent to 500 μl of whole blood at a hematocrit of 40%), or a QIAamp blood midi kit for sample volumes between 200 to 2,000 μl packed red blood cells (Qiagen, Germany). The purified DNA was dried completely in a centrifugal vacuum concentrator and then suspended in a small volume of PCR-grade water to provide a concentrate. The starting volume of whole blood was 1 ml and the purified DNA volume after concentration was 10 μl, so that 1 μl of template suspension corresponds to 100 μl of whole blood. A 2-μl aliquot of the DNA was used as the DNA template in each qPCR. The performance characteristics of this method were therefore assessed for blood sample volumes of >200 μl.

### Quantitative real-time malaria parasite PCR.

The presence of parasites and an estimate of the parasite numbers in each sample were assessed by an absolute quantitative real-time PCR method (Corbett Rotor-Gene Q, Corbett Research, Australia). The 18S rRNA-targeting primers and hydrolysis probes used in the assay were based on a previously published protocol (11), which was modified to improve sensitivity by concentration of the parasite DNA template from large volumes of blood (i.e., 200 μl of packed red cells instead of the conventional 2 μl).

Standard controls were prepared using P. falciparum 3D7 ring-stage synchronized cultured parasites. Suspensions containing precisely 10,000 ring-stage infected red blood cells per tube in packed red blood cells were obtained using fluorescence-activated cell sorting (FACS), following the protocol described by Malleret et al. (2011) (25) using an Influx flow cytometer (Becton, Dickinson). These accurately defined controls were then diluted into carefully measured volumes of white blood cell-depleted red cells obtained from a volunteer with no history of malaria. The samples' DNA were extracted using an automated DNA extraction machine (QIAsymphony and DSP DNA midi kit; Qiagen, Germany) and then dried using a centrifugal vacuum concentrator, and subsequently resuspended in PCR-grade water as described above to yield the DNA concentrate. The standard curve for calibration was made by 5-fold serial dilutions from 250,000, to 16 parasites per milliliter (7 points) with double-distilled water. A QuantiTect multiplex PCR NoROX (Qiagen, Germany) was used, and the PCR mixture was prepared using 1× QuantiTect buffer, 0.4 μM each primer, and 0.2 μM hydrolysis probe. The temperature profiles were 95°C initial denaturation for 15 min, and then 50 cycles of denaturation (94°C) for 15 s followed by annealing (60°C) for 60 s. An internal control was multiplexed in the reaction based on the human beta-actin gene (GenBank accession number, NM_001101.3). The primer and probe sequences were obtained from ShineGene Bio-Technologies, Inc. (primers, 5′-ACCGAGCGCGGCTACAG [forward] and 5′-CTTAATGTCACGCACGATTTCC [reverse]; 6-carboxytetramethylrhodamine [TAMRA] probe, 5′-VIC-TTCACC ACCACGGCCGAG C-3′).

An estimate of the parasite numbers in each sample was derived from extrapolation of the threshold cycle (*C_T_*_)_ value obtained for the unknown sample to the standard curve using the Rotor-Gene Q series software v. 2.0.2. Based on the calibration experiments, the cutoff *C_T_* value was set prospectively to 40. For samples where the qPCR was positive, an attempt was made to determine the Plasmodium species present using nested PCR protocols specific to P. falciparum (microsatellite marker name Pk2), P. vivax (microsatellite marker name 3.502) and Plasmodium malariae (18S rRNA) as described previously (18–20). Positive samples for which there was insufficient DNA to do this or no amplification was obtained were reported as being malaria of indeterminate species.

### qPCR protocol validation.

The technique was validated following published guidelines on diagnostic tests involving nucleic acid amplification and detection (26), and on the minimum information requirements for publication of quantitative real-time PCR experiments (27). The following validation parameters were investigated: analytical sensitivity and specificity, diagnostic sensitivity and specificity, efficiency, precision, analytical and diagnostic accuracies, limit of detection, and robustness. Root cause analysis was conducted for samples with false-positive results. The reference materials used throughout the validation were the samples of accurately known parasite densities obtained through FACS within a range of 20 to 10,000 rings in 100 μl of packed red blood cells (RBCs) as described above.

The sensitivity of our “high-volume” qPCR protocol was also compared to that of the previously published 18S RNA-based protocols; these were the original protocols of Kamau et al. (2011) (11) and Hermsen et al. (2001) (7), and two P. falciparum-specific protocols described by Peradin et al. (2004) (8) and Rougemont et al. (2004) (9) (Table 1). Testing was performed in triplicate reactions for each of the seven FACS reference sample parasite concentrations used to construct the standard calibration control curve (i.e., 250,000, 50,000, 10,000, 2000, 400, 80, and 16 parasites/ml). A QuantiTect multiplex PCR NoROX (Qiagen, Germany) was used, and the PCR mixture and the cycling conditions were per the manufacturer's instructions.

**TABLE 1:** Comparison of the sensitivity of the high-volume qPCR to previously published qPCR protocols

qPCR protocol^*a*^	Rep1/ml^*b*^	Rep2/ml	Rep3/ml	Mean/ml	SD	CV%
Current method	24	43	35.2	34.07	9.55	28.03
Kamau et al. (2011)	655	877	1,353	961.67	356.62	37.08
Hermsen et al. (2001)	8,245	6,573	11,452	8,756.67	2,479.42	28.31
Peradin et al. (2004)	902	1,274	1,543	1,239.67	321.88	25.96
Rougement et al. (2004)	1,220	1,567	2,544	1,777	686.53	38.63

a Kamau et al., reference 11; Hermsen et al. (based on Plasmodium genus specificity), reference 7; Peradin et al., reference 8; Rougemont et al. (based on P. falciparum species specificity), reference 9.

b Each Rep (replicate) column shows the concentration of parasites per ml of blood as determined by each assay. Rep1, Rep 2, and Rep3 represent replicates 1, 2, and 3, respectively.

### Test specificity.

Contamination precautions were rigorously applied in the laboratory. Checking for contamination was performed by routine random insertion of known negative samples (15% of total) in each run. If a negative control yielded a positive result in routine testing, the whole run was repeated and retested until all negative controls gave a negative reading (invariably in the next run). Root cause analysis usually identified the concentrated positive control as the source of contamination. Investigation of potential contamination involved genotyping based on the *msp1*, *msp2*, and *glurp* genes for P. falciparum and on the microsatellite markers pv3.27, pv3.502, and pvms5 for P. vivax, following previously published protocols for P. falciparum (28) and P. vivax (29).

The possibility that cell-free malaria DNA caused false positives was considered. Malaria DNA in merozoites is released from parasitized red cells at the moment of schizont rupture, and free merozoite DNA persists in the circulation. Studies in progress show that plasma DNA concentrations (genomes/μl) are approximately 0.1% of the corresponding whole-blood parasite density (per μl) in acute malaria and are cleared rapidly. Intraleukocytic parasite material is also cleared within days (30). Taken together, these findings indicate that in ultralow parasitemias the extra-erythrocytic DNA is highly unlikely to be a confounder.

In order to rule out cross-contamination of samples during DNA extraction or PCR processing, negative controls consisting of only water were added in a proportion of 8 for every 48 samples.

### Scale-up.

To scale up for high-throughput testing, automated procedures were developed. The samples were labeled using barcodes created from the sample management software Freezerworks (Dataworks Development, Inc., Lynnwood, Seattle, WA). Automated DNA extraction was performed using a QIA Symphony automated nucleic acid extraction integrated system (Qiagen, Germany), and then the QIAgility liquid handling system was used to prepare master mixes for the PCRs and for adding the DNA template.

## RESULTS

### Absolute quantitative real-time PCR malaria parasite density estimation. (i) Quantification calibrators and PCR efficiency.

**FIG 1:**
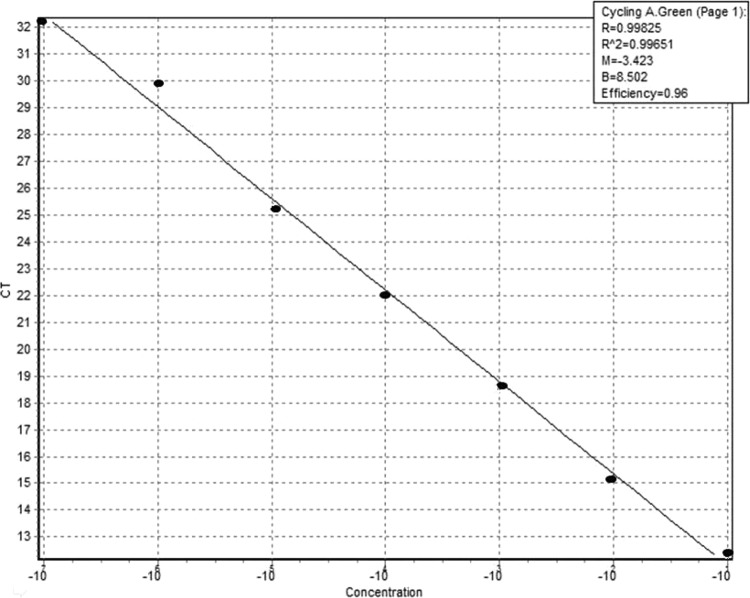
Linearity of serial dilution of standard controls versus the coefficient of determination; *R*^2^ was 0.99651.

The seven reference standards for calibration (250,000, 50,000, 10,000, 2000, 400, 80, and 16 parasites/ml) gave linear standard plots (*R*^2^ > 0.98) with slope values of −3.203 to 3.322 and amplification efficiencies of 90 to 105% (Fig. 1). Nonspecific amplification was not observed during assessment of qPCR products by gel electrophoresis.

### (ii) Analytical sensitivity.

**FIG 2:**
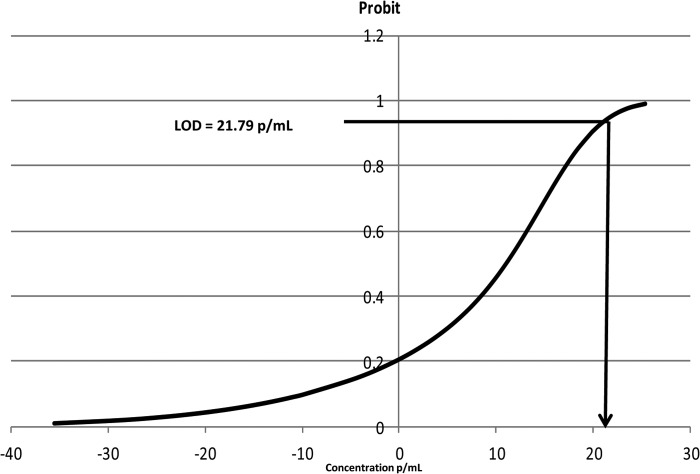
Analytical sensitivity of the malaria qPCR method.

The analytical sensitivity of the high-volume qPCR was derived from the seven-point standard dilution series described above (i.e., from 250,000 to 16 P. falciparum 3D7 ring-stage parasites/ml) analyzed with the Rotor Gene Q (Qiagen, Germany). The experiments were performed on three different days on six replicates. The results were determined by a probit analysis using SPSS 11 (SPSS, Inc., Chicago, IL). A graphical illustration of the probit analysis is shown in Fig. 2. The analytical detection limit (LOD) derived from the plots is 22 (standard deviation [SD], 5) parasites/ml (Fig. 2). This implies that when the assay is applied to the 3D7 parasite strain, there is a 95% probability of detecting a parasitemia as low as 22/ml. Given that the sequence of the target fragment amplified from the P. falciparum 18S RNA genes is exactly the same as that in the 18S RNA genes present in P. vivax, P. ovale, P. malariae, and Plasmodium knowlesi, it is unlikely that the analytical sensitivities for these species would be substantially different than that for P. falciparum. Nonetheless, this would need to be confirmed prospectively.

### (iii) Sensitivity comparison of high-volume qPCR to previously published qPCR protocols.

The sensitivity of the high-volume qPCR protocol was compared to previously published protocols targeting the 18S RNA genes. Two of these are not species specific (7, 11), and two are P. falciparum specific (8, 9). Testing was performed in triplicate for each of the seven-point dilution series (FACS-prepared reference samples) used to obtain the standard calibration plot. Overall, the high-volume qPCR protocol had a mean sensitivity that was 28 to 257 times higher than that of the other methods (Table 1).

### (iv) Diagnostic sensitivity and specificity.

The specificity of the qPCR was assessed in samples obtained from Thai volunteers who had never experienced a malaria infection, and who were not considered to have been exposed to malaria during their lifetime. These samples were also confirmed to be negative for Plasmodium by the reference nested-PCR method targeting the 18S RNA (31). The qPCR assay was negative for all except one sample, presumably a false-positive signal (Table 2). Thus, the diagnostic specificity was 99.75% (95%CI 99.3 to 100%). Twenty one samples from patients with acute malaria confirmed as truly positive for P. falciparum infection using a standard nested PCR species detection technique based on 18S RNA technique (31) were tested and all were positive.

**TABLE 2:** Diagnostic sensitivity and specificity estimates of the malaria qPCR method in method development^*a*^

Result	No. of reference samples	
	Known positive	Known negative
Positive	21 (true positive)	1 (false positive)
Negative	0 (false negative)	400 (true negative)

a Diagnostic sensitivity: true positive (TP)/(TP + false negative [FN]), 100% (21/21); diagnostic sensitivity: TN/(TN + FP), 99.75% (400/401).

### (v) Analytical specificity.

The specificity of the qPCR protocol was tested by three approaches: (i) BLAST analysis of the primers and probe sequences against the NCBI databases, (ii) testing of different field samples harboring the four Plasmodium species that infect humans, and (iii) screening for sequence polymorphisms in the 18S RNA Plasmodium gene fragments amplified.

*(a) BLAST analysis*. The specificity of the qPCR was maximized by the selection of primers and probes highly specific to the Plasmodium genes, which were then used under stringent reaction conditions. The primers and hydrolysis probes selected had already been validated by Kamau et al. (2011) (11), and were shown to have very high specificity for Plasmodium sp. 18S RNA. We conducted updated (9 January 2014) searches using Nucleotide BLAST to confirm that these primers would be unlikely to hybridize and amplify any unrelated genes that have been sequenced recently. There was 100% identity to Plasmodium genus sequences, with no indication that the qPCR might generate positive signals with other organisms, including other parasites in the Plasmodiidae family such as Hepatocystis.

*(b) qPCR testing in field samples of all four human malaria infections*. Blood samples which were microscopy positive for one of four Plasmodium species of humans (100 samples with P. falciparum, 100 samples with P. vivax, 20 samples with P. malariae, and 10 samples with P. ovale) that had been confirmed by the reference method based on nested PCR targeting the 18Ss RNA (31) were tested for specificity. All were positive, as shown in Table 3. Samples from P. knowlesi cases were not available, but sequence analysis predicts that they would also have been positive.

**TABLE 3:** Testing of specificity of the qPCR method on relevant Plasmodium species^*a*^

Plasmodium sp.	No. of samples	No. of positive results
P. falciparum	100	100
P. vivax	100	100
P. malariae	20	20
P. ovale	10	10

a qPCR testing of patient samples harboring one or another of four Plasmodium species that infect humans.

*(c) Screening for sequence polymorphisms*. Direct sequencing of the fragment amplified from more than 70 Plasmodium-positive field samples did not reveal any mutations. Finally, the lack of any bands other than the one expected in the qPCR products amplified from 350 field samples indicated that nonspecific amplification does not occur.

### (vi) Precision.

Precision analysis describes the closeness of agreement between replicate analyses of the same sample. The precision data allow the determination of the total variance of the assay. The total variance consists of the intra-assay variability (variability of multiple results of samples of the same concentration within one experiment) and the interassay variability (variability of multiple results of the assay generated on different instruments of the same type at different times by different operators within the laboratory). The precision data were calculated on the basis of the *C_T_* values of the amplification curves and the parasitemia/ml values. The data obtained were used to determine the standard deviation and the variance.

Assessment of precision was undertaken by testing five of the serially diluted calibration samples (corresponding to 10000, 2000, 400, 80, and 16 parasites/ml, see above) on six separate occasions. The mean coefficient of variation for the cycle threshold across the five samples was 0.78% (Table 4), which translated to a coefficient of variation for parasites/ml of 13.9% (Table 5).

**TABLE 4:** Precision data of the qPCR method on the basis of the qPCR *C_T_* values

Parasite density (p/ml)^*a*^	Mean *C_T_*	*C_T_* SD	*C_T_* coefficient of variation (%)
10,000	30.41	0.25	0.82
2,000	32.55	0.33	1.03
400	34.29	0.24	0.71
80	36.51	0.31	0.85
16	38.83	0.19	0.49
Total variance standard	34.52	0.27	0.78

a p, parasites.

**TABLE 5:** Precision data of the qPCR method on the basis of the parasitemia/ml values

Parasitemia (p/ml)	Mean (p/ml)	SD (p/ml)	Coefficient of variation (%)
10,000	11,005.09	2,243.31	20.38
2,000	2,117.36	277.60	13.11
400	420.17	27.75	6.60
80	99.04	12.53	12.65
16	43.56	7.49	17.19
Total variance standard	2,737.04	513.73	13.99

### (vii) Robustness.

Thirty confirmed Plasmodium-negative blood samples were spiked with 100 parasites/ml of P. falciparum 3D7 control samples. After extraction using the automated DNA extraction machine QIAsymphony and the DSP DNA midi kit (Qiagen, Germany), these samples were analyzed with the qPCR. As a control, an unspiked set of Plasmodium-negative blood samples were processed similarly (*n* = 30). All the control samples were negative, while all the spiked samples were positive, indicating that inhibition of the amplification did not occur. Thus, the robustness of the qPCR is ≥99%.

### (viii) High throughput.

For the high-throughput automated procedures, the capacity for DNA extraction using the QIAsymphony machine (Qiagen, Germany), was 120 samples in 8 h per machine. Concentrating the purified DNA template required 3 h. Resuspension of the template was performed overnight (12 h). The qPCR and nested PCR performed using the QIAgility liquid handling machines took 10 h per machine. These combined procedures allowed us to process 700 blood samples per week, including appropriate checks. The standard manual procedures with the same number of personnel handled a maximum of 200 samples per week.

### (ix) Capillary blood samples on filter paper.

In many field surveys, capillary blood sampling and storage of dried blood on filter paper are performed. This is satisfactory for small blood samples, but with larger volumes drying is slower and the probability of contamination with human or microbial DNA increases. Host white cells are retained in the sample. Drying problems may be countered partially by use of thicker filter paper, although the extraction efficiencies vary between filter paper types. This highly sensitive method is being modified and validated for filter paper, but sensitivities are currently four to five times lower, so the current specifications refer to centrifuged whole blood samples only.

## DISCUSSION

This study describes a highly sensitive and specific qPCR method using a relatively large blood volume and enabling accurate assessment of malaria parasite densities as low as 22 parasites/ml of blood. The method is based on existing protocols (11) using simple, but essential, modifications: the use of a higher blood volume results in a larger amount of Plasmodium DNA which can be processed. However, host leukocyte DNA concentrations will also be increased concomitantly, so that the careful removal of the buffy coat from the centrifuged sample is a prerequisite to avoid interference during PCR processing. The larger blood sample volumes require venipuncture, since capillary blood sampling of >200 μl from a finger prick sample is difficult. In addition, a finger prick sample often leads to interstitial fluid dilution as the finger is squeezed, and the host white cells (and thus host DNA) cannot be removed. If blood-collecting tubes are not used, then thick filter papers are required with variable DNA extraction efficiencies, and incomplete drying increases the risks of contamination and microbial overgrowth.

The high-volume method was shown to be robust, reliable, and accurate. The very high sensitivity of the method demands that scrupulous care is taken to avoid contamination. To this end, routine inclusion of standard low-density positive as well as negative controls in sample series, preferably blinded, is strongly recommended. Calibration and quality assurance would be facilitated by global quality assurance schemes similar in operations to those instituted for antimalarial drugs (World Wide Antimalarial Resistance Network [WWARN] [32]). An important practical application of the ultrasensitive high-throughput method is in malaria epidemiological surveys to determine the asymptomatic Plasmodium burden in a population in low-transmission settings. The methodology presented here is now being applied to samples collected during ongoing studies to define the epidemiology of malaria in different regions of South-East Asia and to plan elimination strategies. Preliminary results indicate high rates of asymptomatic low-density parasite carriage in some areas. Conventional microscopy or rapid diagnostic tests typically detect parasite densities reliably down to 50,000 to 100,000 parasites/ml, whereas capillary blood filter paper PCR methods, usually sampling 5 μl of whole blood, will detect densities down to around 100 parasites per ml. The exact relationship between these different detection methods with different detection limits needs to be determined and reflects the shape of the parasite density distributions in different populations under different epidemiological circumstances. If there is a predictable relationship between the less sensitive methods and this high-volume method (33), capillary blood filter paper methods might be a useful surrogate measure for the true Plasmodium prevalence in a population. Appropriate filter paper and storage methods will then need to be assessed and validated. However, it is likely that the more sensitive methods will be needed in an elimination setting in which malaria transmission is reduced to low levels.

Some additional factors may warrant consideration when interpreting the results, given the high sensitivity of the qPCR protocol described here. First, it is possible that malaria DNA released from dead parasites will give rise to a positive result, strictly speaking a false positive. This depends on the amount released and the time for which such DNA persists in the circulation. Data from clinical studies indicate that free DNA would be a significant confounder only if recent parasitemias were high (well into the range detectable by microscopy) and if the sample from which the DNA was purified contained >10% plasma. This is unlikely to pose a problem for the qPCR protocol if the plasma is removed, particularly in low-transmission settings where microscopy-positive parasitemia (i.e., >50,000 parasites/ml) is likely to be associated with clinical signs. Second, the liberation of large numbers of merozoites from the hepatic schizonts (typically 10,000 to 30,000 merozoites from each hepatic schizont) might lead to the circulation of ∼300,000 merozoites following synchronous rupture of 10 hepatic forms (the median sporozoite inoculum is considered to be around 10), i.e., ∼60 parasites/ml (a higher density in children who have lower blood volumes), which would be likely to give a positive result for the qPCR protocol (sensitivity of ∼20 parasites/ml). In areas of low transmission intensity (<10 infectious bites per year), the likelihood of sampling at the exact time of hepatic schizont rupture is low. In higher-transmission settings where most infections are self-limiting this might be a significant confounder. Finally, for the parasite species where the mature forms do not sequester significantly (non-P. falciparum), a maturing erythrocytic schizont might contain up to 24 parasite genomes, which would overestimate the parasitemia estimated by the qPCR method.

In conclusion, we describe a highly sensitive and specific high-volume qPCR method that allows assessment of the asymptomatic reservoir of parasitemic individuals with chronic very low-level Plasmodium infections, thereby providing an accurate assessment of the malaria epidemiology. The utility of this new method will now require further evaluation in diverse malaria endemic settings, and in particular those striving for malaria elimination.

## Supplementary Material

Supplemental material

## References

[1] Dondorp AM Nosten F Yi P Das D Phyo AP Tarning J Lwin KM Ariey F Hanpithakpong W Lee SJ Ringwald P Silamut K Imwong M Chotivanich K Lim P Herdman T An SS Yeung S Singhasivanon P Day NP Lindegardh N Socheat D White NJ 2009 Artemisinin resistance in Plasmodium falciparum malaria. N. Engl. J. Med. 361:455–467. 10.1056/NEJMoa0808859. 19641202 PMC3495232

[2] Maude RJ Pontavornpinyo W Saralamba S Aguas R Yeung S Dondorp AM Day NP White NJ White LJ 2009 The last man standing is the most resistant: eliminating artemisinin-resistant malaria in Cambodia. Malar. J. 8:31. 10.1186/1475-2875-8-31. 19228438 PMC2660356

[3] White NJ 2010 Artemisinin resistance—the clock is ticking. Lancet 376:2051–2052. 10.1016/S0140-6736(10)61963-0. 21168039

[4] Snounou G Viriyakosol S Jarra W Thaithong S Brown KN 1993 Identification of the four human malaria parasite species in field samples by the polymerase chain reaction and detection of a high prevalence of mixed infections. Mol. Biochem. Parasitol. 58:283–292. 10.1016/0166-6851(93)90050-8. 8479452

[5] Imoukhuede EB Andrews L Milligan P Berthoud T Bojang K Nwakanma D Ismaili J Buckee C Njie F Keita S Sowe M Lang T Gilbert SC Greenwood BM Hill AV 2007 Low-level malaria infections detected by a sensitive polymerase chain reaction assay and use of this technique in the evaluation of malaria vaccines in an endemic area. Am. J. Trop. Med. Hyg. 76:486–493. 17360872 PMC3836239

[6] Okell LC Ghani AC Lyons E Drakeley CJ 2009 Submicroscopic infection in Plasmodium falciparum-endemic populations: a systematic review and meta-analysis. J. Infect. Dis. 200:1509–1517. 10.1086/644781. 19848588

[7] Hermsen CC Telgt DS Linders EH van de Locht LA Eling WM Mensink EJ Sauerwein RW 2001 Detection of Plasmodium falciparum malaria parasites *in vivo* by real-time quantitative PCR. Mol. Biochem. Parasitol. 118:247–251. 10.1016/S0166-6851(01)00379-6. 11738714

[8] Perandin F Manca N Calderaro A Piccolo G Galati L Ricci L Medici MC Arcangeletti MC Snounou G Dettori G Chezzi C 2004 Development of a real-time PCR assay for detection of Plasmodium falciparum, Plasmodium vivax, and Plasmodium ovale for routine clinical diagnosis. J. Clin. Microbiol. 42:1214–1219. 10.1128/JCM.42.3.1214-1219.2004. 15004078 PMC356834

[9] Rougemont M Van Saanen M Sahli R Hinrikson HP Bille J Jaton K 2004 Detection of four Plasmodium species in blood from humans by 18S rRNA gene subunit-based and species-specific real-time PCR assays. J. Clin. Microbiol. 42:5636–5643. 10.1128/JCM.42.12.5636-5643.2004. 15583293 PMC535226

[10] Schneider P Wolters L Schoone G Schallig H Sillekens P Hermsen R Sauerwein R 2005 Real-time nucleic acid sequence-based amplification is more convenient than real-time PCR for quantification of Plasmodium falciparum. J. Clin. Microbiol. 43:402–405. 10.1128/JCM.43.1.402-405.2005. 15635001 PMC540116

[11] Kamau E Tolbert LS Kortepeter L Pratt M Nyakoe N Muringo L Ogutu B Waitumbi JN Ockenhouse CF 2011 Development of a highly sensitive genus-specific quantitative reverse transcriptase real-time PCR assay for detection and quantitation of plasmodium by amplifying RNA and DNA of the 18S rRNA genes. J. Clin. Microbiol. 49:2946–2953. 10.1128/JCM.00276-11. 21653767 PMC3147742

[12] Beshir KB Hallett RL Eziefula AC Bailey R Watson J Wright SG Chiodini PL Polley SD Sutherland CJ 2010 Measuring the efficacy of anti-malarial drugs in vivo: quantitative PCR measurement of parasite clearance. Malar. J. 9:312. 10.1186/1475-2875-9-312. 21054863 PMC2992070

[13] Hwang J Jaroensuk J Leimanis ML Russell B McGready R Day N Snounou G Nosten F Imwong M 2012 Long-term storage limits PCR-based analyses of malaria parasites in archival dried blood spots. Malar. J. 11:339. 10.1186/1475-2875-11-339. 23043522 PMC3507721

[14] Farrugia C Cabaret O Botterel F Bories C Foulet F Costa JM Bretagne S 2011 Cytochrome *b* gene quantitative PCR for diagnosing Plasmodium falciparum infection in travelers. J. Clin. Microbiol. 49:2191–2195. 10.1128/JCM.02156-10. 21508150 PMC3122771

[15] Campino S Auburn S Kivinen K Zongo I Ouedraogo JB Mangano V Djimde A Doumbo OK Kiara SM Nzila A Borrmann S Marsh K Michon P Mueller I Siba P Jiang H Su XZ Amaratunga C Socheat D Fairhurst RM Imwong M Anderson T Nosten F White NJ Gwilliam R Deloukas P MacInnis B Newbold CI Rockett K Clark TG Kwiatkowski DP 2011 Population genetic analysis of Plasmodium falciparum parasites using a customized Illumina GoldenGate genotyping assay. PLoS One 6:e20251. 10.1371/journal.pone.0020251. 21673999 PMC3108946

[16] Berry A Deymier C Sertorio M Witkowski B Benoit-Vical F 2009 Pfs 16 pivotal role in Plasmodium falciparum gametocytogenesis: a potential antiplasmodial drug target. Exp. Parasitol. 121:189–192. 10.1016/j.exppara.2008.10.010. 19014941

[17] Mercereau-Puijalon O Barale JC Bischoff E 2002 Three multigene families in Plasmodium parasites: facts and questions. Int. J. Parasitol. 32:1323–1344. 10.1016/S0020-7519(02)00111-X. 12350369

[18] Alemayehu S Feghali KC Cowden J Komisar J Ockenhouse CF Kamau E 2013 Comparative evaluation of published real-time PCR assays for the detection of malaria following MIQE guidelines. Malar. J. 12:277. 10.1186/1475-2875-12-277. 23927553 PMC3750446

[19] Andrews L Andersen RF Webster D Dunachie S Walther RM Bejon P Hunt-Cooke A Bergson G Sanderson F Hill AV Gilbert SC 2005 Quantitative real-time polymerase chain reaction for malaria diagnosis and its use in malaria vaccine clinical trials. Am. J. Trop. Med. Hyg. 73:191–198. 16014857

[20] Wang B Han SS Cho C Han JH Cheng Y Lee SK Galappaththy GN Krongthong T Soe MT Oo HW Kyaw MP Han ET 2014 Comparison of microscopy, nested and real-time PCR-based assays with high-throughput pooled samples for screening asymptomatic malaria carriers from endemic areas of Myanmar. J. Clin. Microbiol. 52:1838–1845. 10.1128/JCM.03615-13. 24648557 PMC4042795

[21] Starzengruber P Fuehrer HP Ley B Thriemer K Swoboda P Habler VE Jung M Graninger W Khan WA Haque R Noedl H 2014 High prevalence of asymptomatic malaria in south-eastern Bangladesh. Malar. J. 13:16. 10.1186/1475-2875-13-16. 24406220 PMC3896725

[22] Golassa L Enweji N Erko B Aseffa A Swedberg G 2013 Detection of a substantial number of submicroscopic Plasmodium falciparum infections by polymerase chain reaction: a potential threat to malaria control and diagnosis in Ethiopia. Malar. J. 12:352. 10.1186/1475-2875-12-352. 24090230 PMC3850638

[23] Ganguly S Saha P Guha SK Biswas A Das S Kundu PK Maji AK 2013 High prevalence of asymptomatic malaria in a tribal population in eastern India. J. Clin. Microbiol. 51:1439–1444. 10.1128/JCM.03437-12. 23426929 PMC3647920

[24] Hoyer S Nguon S Kim S Habib N Khim N Sum S Christophel EM Bjorge S Thomson A Kheng S Chea N Yok S Top S Ros S Sophal U Thompson MM Mellor S Ariey F Witkowski B Yeang C Yeung S Duong S Newman RD Menard D 2012 Focused screening and treatment (FSAT): a PCR-based strategy to detect malaria parasite carriers and contain drug resistant P. falciparum, Pailin, Cambodia. PLoS One 7:e45797. 10.1371/journal.pone.0045797. 23049687 PMC3462177

[25] Malleret B Claser C Ong AS Suwanarusk R Sriprawat K Howland SW Russell B Nosten F Renia L 2011 A rapid and robust tri-color flow cytometry assay for monitoring malaria parasite development. Sci. Rep. 1:118. 10.1038/srep00118. 22355635 PMC3216599

[26] Saunders N Zambon M Sharp I Siddiqui R Bermingham A Ellis J Vipond B Sails A Moran-Gilad J Marsh P Guiver M Division HPAMS 2013 Guidance on the development and validation of diagnostic tests that depend on nucleic acid amplification and detection. J. Clin. Virol. 56:260–270. 10.1016/j.jcv.2012.11.013. 23246357

[27] Bustin SA Benes V Garson JA Hellemans J Huggett J Kubista M Mueller R Nolan T Pfaffl MW Shipley GL Vandesompele J Wittwer CT 2009 The MIQE guidelines: minimum information for publication of quantitative real-time PCR experiments. Clin. Chem. 55:611–622. 10.1373/clinchem.2008.112797. 19246619

[28] Anderson TJ Su XZ Bockarie M Lagog M Day KP 1999 Twelve microsatellite markers for characterization of Plasmodium falciparum from finger-prick blood samples. Parasitology 119(Pt 2):113–125. 10466118 10.1017/s0031182099004552

[29] Imwong M Nair S Pukrittayakamee S Sudimack D Williams JT Mayxay M Newton PN Kim JR Nandy A Osorio L Carlton JM White NJ Day NP Anderson TJ 2007 Contrasting genetic structure in Plasmodium vivax populations from Asia and South America. Int. J. Parasitol. 37:1013–1022. 10.1016/j.ijpara.2007.02.010. 17442318

[30] Day NP Pham TD Phan TL Dinh XS Pham PL Ly VC Tran TH Nguyen TH Bethell DB Nguyan HP White NJ 1996 Clearance kinetics of parasites and pigment-containing leukocytes in severe malaria. Blood 88:4694–4700. 8977263

[31] Snounou G Singh B 2002 Nested PCR analysis of Plasmodium parasites. Methods Mol. Med. 72:189–203. 10.1385/1-59259-271-6:189. 12125116

[32] Lourens C Watkins WM Barnes KI Sibley CH Guerin PJ White NJ Lindegardh N 2010 Implementation of a reference standard and proficiency testing programme by the World Wide Antimalarial Resistance Network (WWARN). Malar. J. 9:375. 10.1186/1475-2875-9-375. 21184684 PMC3020159

[33] Okell LC Bousema T Griffin JT Ouedraogo AL Ghani AC Drakeley CJ 2012 Factors determining the occurrence of submicroscopic malaria infections and their relevance for control. Nat. Commun. 3:1237. 10.1038/ncomms2241. 23212366 PMC3535331

