# Supplementary material for: High-Throughput Ultrasensitive Molecular Techniques for Quantifying Low-Density Malaria Parasitemias
Source: J Clin Microbiol. 2014 Sep;52(9):3303–9. doi: 10.1128/JCM.01057-14 (PMC4313154; doi:10.1128/JCM.01057-14)
Supplement: Supplemental material [file supp_52_9_3303__index.html]

High-Throughput Ultrasensitive Molecular Techniques for Quantifying Low-Density Malaria Parasitemias — Supplemental material 

# High-Throughput Ultrasensitive Molecular Techniques for Quantifying Low-Density Malaria Parasitemias

## Supplemental material

**Files in this Data Supplement:**

- Supplemental file 1 -

  Table S1 (Probit analysis of the standard dilution series)

  PDF, 61K
