# Supplementary material for: High-Throughput Ultrasensitive Molecular Techniques for Quantifying Low-Density Malaria Parasitemias
Source: J Clin Microbiol. 2014 Sep;52(9):3303–9. doi: 10.1128/JCM.01057-14 (PMC4313154; doi:10.1128/JCM.01057-14)
Supplement: Supplemental material [file JCM.01057-14_zjm999093684so1.pdf]

Supplementary Table 1: Probit analysis of the standard dilution series

		95% Confidence Limits (DNA copies equivalence/mL)		
		Estimate	Lower Bound	Upper Bound
PROBIT	.010	-1.113	-35.542	6.642
	.020	1.082	-28.551	8.273
	.030	2.475	-24.176	9.368
	.040	3.522	-20.927	10.233
	.050	4.375	-18.316	10.970
	.060	5.100	-16.123	11.625
	.070	5.736	-14.225	12.225
	.080	6.306	-12.548	12.785
	.090	6.823	-11.045	13.316
	.100	7.300	-9.682	13.826
	.150	9.274	-4.298	16.195
	.200	10.843	-.414	18.471
	.250	12.189	2.561	20.782
	.300	13.398	4.918	23.172
	.350	14.518	6.836	25.653
	.400	15.580	8.438	28.224
	.450	16.609	9.816	30.885
	.500	17.620	11.033	33.642
	.550	18.632	12.140	36.510
	.600	19.661	13.173	39.514
	.650	20.723	14.166	42.696
	.700	21.843	15.146	46.115
	.750	23.052	16.144	49.863
	.800	24.398	17.200	54.093
	.850	25.967	18.374	59.080
	.900	27.941	19.788	65.418
	.910	28.417	20.121	66.958
	.920	28.935	20.480	68.633
	.930	29.505	20.871	70.479
	.940	30.141	21.304	72.544
	.950	30.866	21.793	74.904

	.960	31.719	22.362	77.682
	.970	32.766	23.054	81.105
	.980	34.159	23.963	85.665
	.990	36.354	25.376	92.874
